# CD8^+^ stem cell-like memory T cells: Unveiling the potential for next-generation vaccination strategies

**DOI:** 10.1016/j.xcrm.2026.102990

**Published:** 2026-08-18

**Authors:** Laury Nguema, Yves Lévy, Véronique Godot

**Affiliations:** 1 Faculté de Santé, Université Paris-Est, Créteil, France; 2 INSERM U955, Team Accelerates, Institut Mondor de Recherche Biomédicale (IMRB), Créteil, France; 3 Vaccine Research Institute (VRI), Créteil, France; 4 Service d'Immunologie Clinique, Groupe Henri-Mondor Albert-Chenevier, Assistance Publique-Hôpitaux de Paris, Créteil, France; 5 IHU SEPSIS - PROMETHEUS Comprehensive Sepsis Center, Paris-Saclay University, Saclay, France

**Keywords:** CD8^+^ Tscm cells, self-renewal properties, memory T cells, vaccination

## Abstract

The fields of vaccinology and tumor immunology have long prioritized CD8^+^ central memory T (Tcm) cells as markers of long-term protection, often neglecting the cell population responsible for the most durable immune memory. Recent advances in T cell biology have clearly established the existence of stem cell-like memory T (Tscm) lymphocytes, now regarded as major emerging contributors. CD8^+^ Tscm cells are highlighted for their capacity for long-term self-renewal, multipotency, and robust recall responses, features that make them attractive targets in chronic infection and cancer immunotherapy. The real revolution is mechanistic: Tscm cell differentiation follows a defined blueprint integrating a specific metabolic profile, lymphoid niche control, and the T cell receptor (TCR) “Goldilocks principle,” tuned by costimulatory networks. This review synthesizes current mechanistic knowledge of Tscm cell induction and discusses how it can guide the rational design of future vaccines and adoptive cell therapies engineered to generate these long-lived populations.

## Graphical abstract

**Figure:**
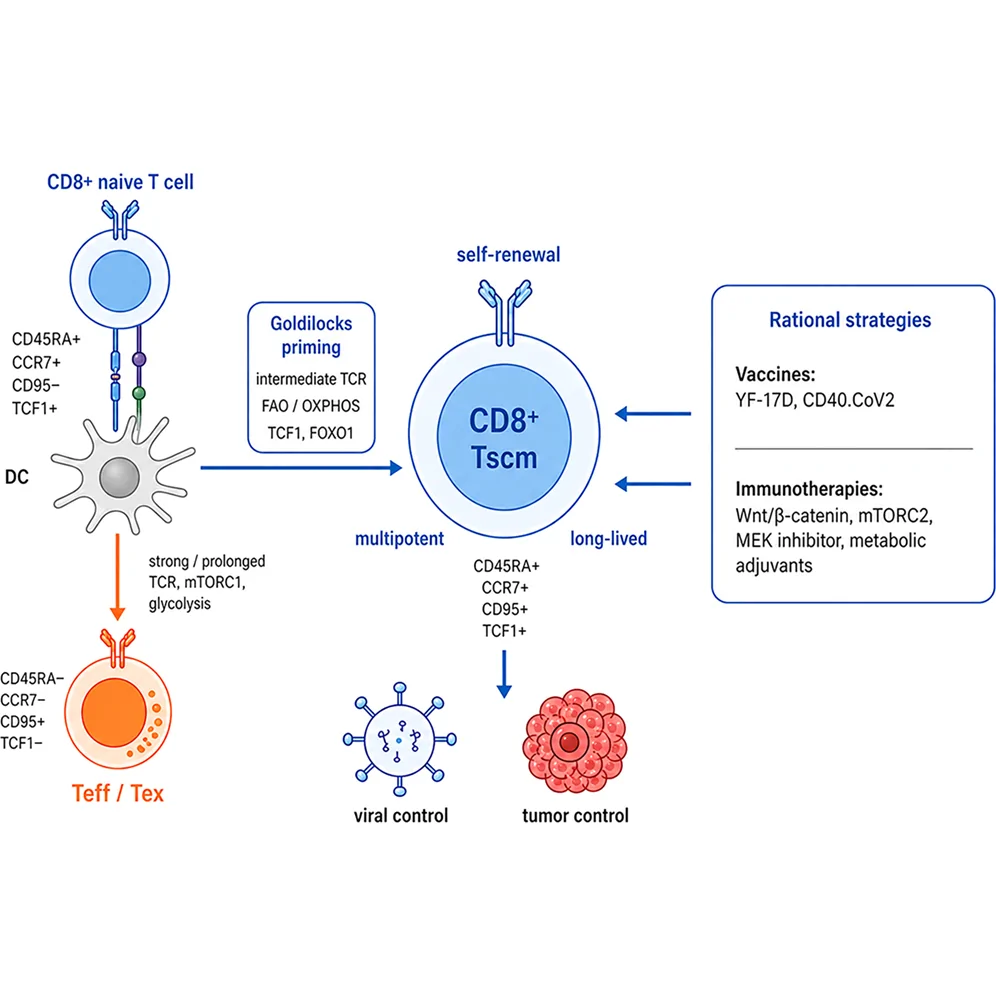

## Teaser

In this review, Nguema et al. define CD8^+^ stem cell-like memory T cells as key mediators of durable CD8^+^ immunity in viral infections and cancer and distill a mechanistic blueprint for next-generation vaccines and adoptive T cell therapies deliberately engineered to generate long-lived Tscm cell populations.

## Introduction

Adaptive immunity relies on the generation and maintenance of memory T cells to provide long-term protection against antigen re-exposure. The traditional view of T cell memory has been built around the circulating central memory T (Tcm) cell, effector memory T (Tem) cell, and terminally differentiated effector memory (Temra) subsets, with Tcm considered as a major source of Tem during recall responses^1^ and the tissue-resident memory T cells seeded in mucosal sites and in the skin.^2^ This framework was further expanded by the 2005 discovery of stem cell-like memory T (Tscm) cells in mouse models of graft-versus-host disease.^3^ Therefore, a new concept emerges, suggesting that T cell memory depends on a stem cell-like, self-renewing population that persists without antigen re-exposure and can differentiate into memory T cells.^4^ These cells can persist for decades, as demonstrated by their maintenance following yellow fever (YF) vaccination for over 25 years.^5^ Tscm cells bridge stem cell biology and immunological memory^4^ and exhibit three defining features: self-renewal capacity, multipotency, and exceptional longevity. Rather than simply being “less differentiated” memory cells, Tscm cells are better characterized as hybrid cells that combine naive-like and memory-like features at multiple levels. Phenotypically and transcriptionally, Tscm cells retain core naive traits while already expressing hallmark memory-associated markers and gene programs. Epigenetic profiling of long-lived human CD8^+^ memory cells points to an effector-like open chromatin landscape, supporting the view that Tscm cells integrate naive-like and memory-like epigenetic features.^6^^,^^7^ Because of their multipotency, Tscm cells rapidly generate all memory and effector types upon antigen re-exposure, providing both immediate protection and sustained immune memory. For therapeutic and vaccination strategies alike, inducing and developing antigen-specific Tscm cells remains a critical hurdle to improving long-term outcomes against cancer and infections. Indeed, vaccines capable of generating Tscm cell responses could protect for decades without needing recall injections. However, to achieve this goal, we must understand the molecular mechanisms governing Tscm cell generation and develop rational strategies to exploit these pathways for therapeutic purposes. This review introduces the phenotypic and functional characteristics of CD8^+^ Tscm cells and their roles in human diseases, with a focus on viral infections and cancers. We outline the molecular and environmental factors involved in the induction of CD8^+^ Tscm cells and discuss vaccination strategies and next-generation approaches for generating CD8^+^ Tscm cells.

## Tscm cell biological foundation and regulatory mechanisms

The strong potential of CD8^+^ Tscm cells in durable immunity rests on their phenotypic, functional, and metabolic characteristics, some of which are shared with Tcm cells. Indeed, a single-cell lineage-tracing study in mice reported that a significant fraction of Tcm cells isolated 100 or even 500 days after an acute infection retained sufficient proliferative and differentiation potential to fully reconstitute immunocompetence in RAG-deficient hosts and to mediate protection upon secondary challenge.^8^ These data illustrate that the relative contribution of Tscm cells to long-term recall responses can vary with species, infection model, and the phenotypic criteria used to distinguish Tscm cells from Tcm cells. They also support a nuanced view in which stem-like memory functions are enriched in, but not necessarily exclusive to, the Tscm cell subset. From a therapeutic perspective, clarifying how molecular and environmental cues sustain Tscm cell features within this broader CD8^+^ memory landscape will be essential to selectively harness the most durable and versatile memory pools.

### Phenotypic and functional identity: The stem cell-memory hybrid

The identification of CD8^+^ Tscm cells across species relies on a combination of phenotypic markers, many of which are shared with other memory T cell subsets or naive T cells (Table 1). In humans and non-human primates, they are defined by a specific surface marker profile: CD45RA^+^ CD62L^+^ CD95^+^ CD27^+^ CD28^+^ CCR7^+^ CD122^+^ CD127^+^ CXCR3^+^.^4^^,^^9^ This distinguishes them from CD95-negative naive T cells and more differentiated memory subsets. Murine Tscm cells exhibit a slightly different phenotype, identified as CD44^lo^ CD62L^+^ Sca-1^+^ CCR7^+^ CXCR3^+^ CD122,^3^^,^^10^ with Sca-1 serving as a key stemness marker. CD8^+^ Tscm and other stem-like CD8^+^ T cell subsets in mice and humans express high levels of the transcription factor TCF1 (*Tcf7*).^11^^,^^12^ Functional studies in *Tcf7*^hi^ stem-like precursors have shown that TCF1 is required for their long-term self-renewal, recall potential, and capacity to generate central memory CD8^+^ T cells.^11^^,^^12^ Beyond stem-like CD8^+^ subsets, TCF1 is likewise required for the maintenance and recall capacity of conventional central memory CD8^+^ T cells, further underscoring its central role in memory T cell biology.^13^ CD8^+^ Tscm cells display functional properties characteristic of other stem-like or central memory precursors, but at a higher level of self-renewal and multipotency. Asymmetric cell division, described in early CD8^+^ T cell fate studies, supports self-renewal and the generation of less- and more differentiated progeny across memory precursors^14^^,^^15^^,^^16^^,^^17^; yet, single-cell-derived Tscm cells regenerate the full spectrum of memory and effector subsets over repeated rounds of antigenic challenge, whereas conventional Tcm cells predominantly act as progenitors of Tem/T effector (Teff) cells during recall responses.^11^^,^^18^

**Table 1:** Phenotypic markers of CD8^+^ Tscm and other memory T cells

Marker	Tscm cells		Tpex cells		Tcm cells		Tem cells		Teff cells	
	Human/NHP	Mouse	Human/NHP	Mouse	Human/NHP	Mouse	Human/NHP	Mouse	Human/NHP	Mouse
CD45RA/CD44	CD45RA+^a^	CD44lo	CD45RA	CD44lo	CD45RA−	CD44^+^	CD45RA−	CD44^+^	CD45RA−	CD44^+^
CD62L	+	+	+	+	+	+	−	−	−	−
CCR7	+	+	+	+	+	+	−	−	−	−
CD95	+	+	+	+	+	+	+	+	+	+
CD27/CD28	+	variable	+	+	+	NR	+	NR	−	NE
Sca-1	+	+	NR	NR	NR	NR	NR	NR	NR	NR
CD122	+	+	+	+	+	NR	+	NR	+	NR
CD127	+	BES-ND	+	+	+	+	+^b^	BES-ND	−	−
CD11a	+	NR	NR	NR	+	NR	+	NR	+	NR
CD55	BES-ND	BES-ND	−	−	BES-ND	BES-ND	BES-ND	BES-ND	BES-ND	BES-ND
CXCR3	+	+	+	+	+	NR	+	NR	+	+
CXCR4	+	−	+	+	+	NR	+	NR	+	NR
CXCR5	NR	NR	+	+	NR	NR	NR	NR	NR	NR
TCF1	+	+	+	+	+	+	+	+	−	−
T-bet	−	−	−	−	+	NR	+	NR	+	+
TOX	−	−	+	+	−	−	−	−	−	−
KLF2	BES-ND	BES-ND	+	+	BES-ND	BES-ND	BES-ND	BES-ND	−	−
Bcl-2	+	+	+	+	+	NR	+	NR	+	NR
KLRG1	−	−	−	−	−	−	+	+	+	+
SLAMF6	+BES	+BES	+	+	+BES	+BES	+BES	+BES	+BES	+BES
PD-1	−	−	+	+	−	−	−	−	−	−
TIM3	−	−	−	−	−	−	−	−	−	−
TIGIT	−	−	+	+	−	−	−	−	−	−

NR, not reported; NHP, non-human primate; Tscm cells, stem cell-like memory T cells; Tcm cells, central memory T cells; Tem cells, effector memory T cells; Teff cells, effector T cells.

BES-ND, broadly expressed by memory T cells; subset-specific expression not defined.

+BES, SLAMF6 is broadly expressed and often upregulated on non-naive human CD8^+^ T cells, including central memory, effector, and stem-like/progenitor-exhausted subsets; it can help enrich for stem-like populations in defined settings but does not discriminate classical Tscm from other memory or effector cells.

a CD45RA is not an exclusive or definitive stem-like marker, as it can be shared by naive, Tscm, and Temra/late effector CD8 T cells.

b CD127 may be transiently downregulated in the context of chronic activation.

Functionally, CD8^+^ Tscm cells can mount a type 1 cytokine response after restimulation; however, their immediate effector output is modest. They generally produce lower levels of interferon (IFN)-γ and TNF and exhibit weaker cytotoxic activity compared with conventional Tcm cells, and even more so than Tem cells (Tem/Teff).^4^ Nonetheless, Tscm cells maintain a relatively strong IL-2 response, which reflects their less-differentiated and more proliferative profile. Granzyme B expression is low or undetectable at baseline and remains lower than that of Tcm and Tem/Teff cells after activation. Indeed, single-cell RNA sequencing datasets indicate that CD8^+^ Tscm cells have few cytotoxic transcripts. In contrast, ATAC-seq and other epigenetic analyses reveal open chromatin at key effector loci (*IFNG, GZMB*, and *PRF1*), suggesting that these cells are poised to acquire full cytotoxic function when appropriately stimulated.^4^^,^^6^^,^^9^^,^^19^^,^^20^ Importantly, they maintain low baseline metabolic activity and take longer to become exhausted than other memory T cells, thereby better preserving their long-term functionality.^21^ The longevity of Tscm cells is supported by enhanced anti-apoptotic molecule expression (Bcl-2), efficient DNA repair, and specific metabolic programs that minimize oxidative stress.^4^^,^^22^

As mentioned earlier, a persistent challenge in the Tscm cell field is the absence of a singular, specific surface marker or transcriptional signature that unambiguously identifies this subset. Current definitions rely on combinations of markers that are largely shared with other memory populations, making it difficult to compare studies and to fully apprehend Tscm biology across different settings. It is, therefore, often unclear whether published data consistently refer to the same pool of memory precursors defined by non-identical phenotypic criteria or whether they instead capture genuinely heterogeneous populations within the stem cell-like memory compartment. While such putative subsets remain difficult to delineate by phenotype alone, kinetic and mathematical modeling of human vaccine-induced antigen-specific CD8^+^ T cells reveals an underlying kinetic heterogeneity of the memory pool, with at least two components, a small but very long-lived fraction (9-year half-life) and a more abundant, shorter-lived fraction with higher turnover (5 months), together sustaining protective immunity over many years.^23^ Concurrently, research in chronic infection and cancer has identified a population of TCF1^+^ PD-1^+^ “progenitor-exhausted” CD8^+^ T cells (Table 1) that emerge prior to terminal exhaustion.^24^^,^^25^^,^^26^ Phenotypically and transcriptionally, these cells occupy a stem-like, less-differentiated state rather than a terminally exhausted state.^25^ Slamf6^+^ TCF1^+^ PD-1^+^ cells maintain productive CD8^+^ T cell responses under persistent antigen exposure, exhibit self-renewal capacity, and continuously give rise to more differentiated exhausted progeny.^24^ Their transcriptional profile remains relatively naive like compared with fully exhausted cells, capturing several features of stem-like memory, while being specifically programmed by sustained antigen and co-inhibitory signaling. Rather than representing classical Tscm diverted toward exhaustion, these cells are more accurately characterized as a distinct stem-like adaptation to chronic infection or cancer. In contrast, during acute-resolving conditions, early antigen-experienced precursors transiently upregulate PD-1 at the peak of the response and downregulate it as antigen is cleared, promoting a conventional Tscm- or central memory-like trajectory.^12^^,^^24^^,^^25^^,^^26^

### Conceptual models of stem-like CD8^+^ memory (including Tscm cell) differentiation

The position of Tscm cells within the broader landscape of CD8^+^ T cell differentiation is still a matter of debate, with several non-mutually exclusive models proposed to explain how stem cell-like memory arises from naive precursors.

**Figure 1:**
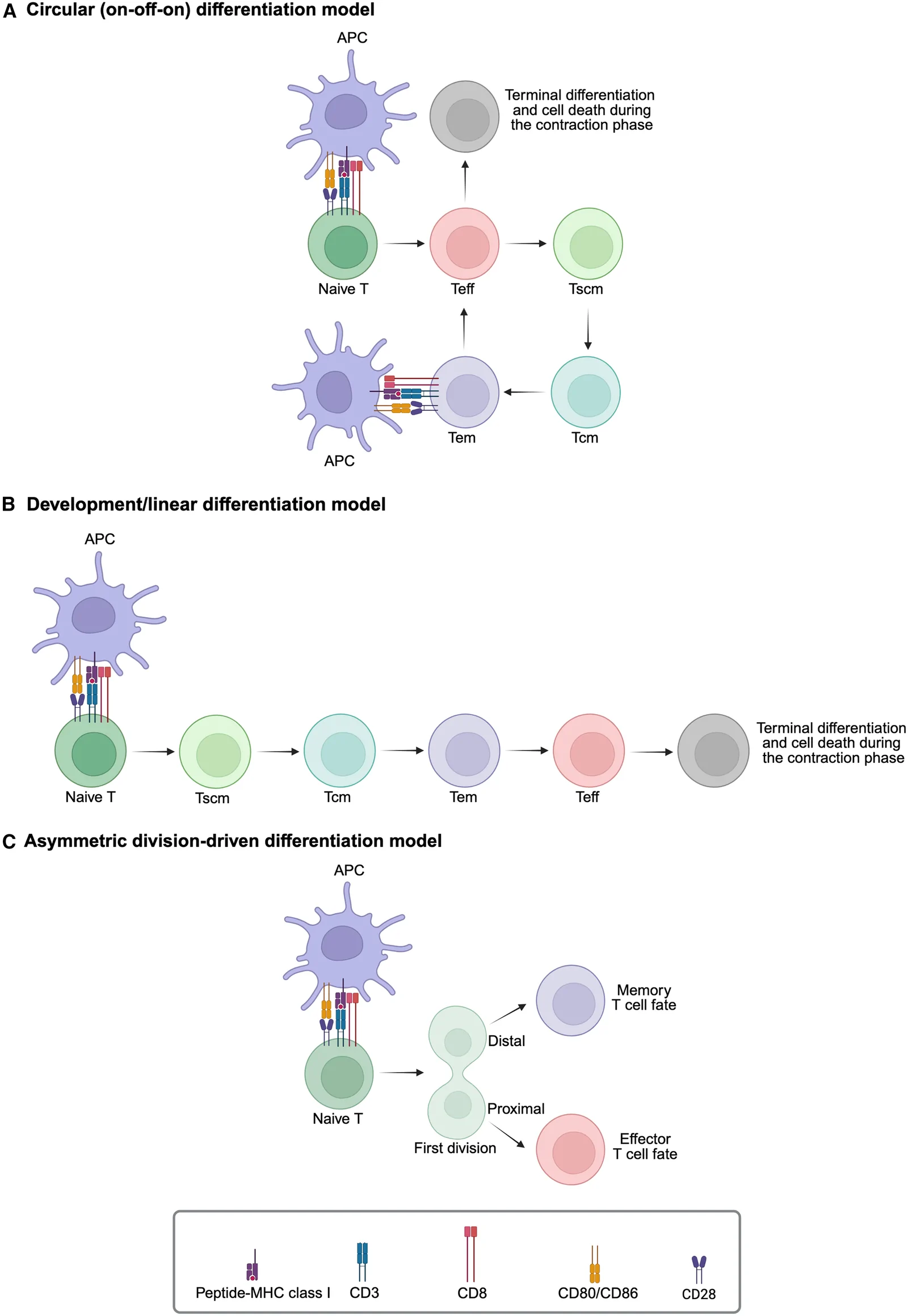
Three hypothetical models of CD8^+^ T cell differentiation and positioning of Tscm cells (A) In the circular (on-off-on) differentiation model, activated T cells first acquire effector functions and then dedifferentiate back into Tscm or other memory subsets, before some cells undergo terminal differentiation and die during the contraction phase. (B) In the developmental/linear differentiation model, activated naive T cells progressively lose stem-like and memory properties and acquire effector functions along a unidirectional trajectory (Tscm → Tcm → Tem → Teff), with the degree of differentiation influenced by the strength and duration of TCR and co-stimulatory signals. (C) In the asymmetric division-driven differentiation model, the first division of an activated naive T cell gives rise to two daughter cells with divergent fates: a proximal daughter cell (closer to the APC) that preferentially adopts an effector T cell fate and a distal daughter cell (further from the APC) that preferentially adopts a memory-biased fate, from which Tscm and other memory subsets may arise.

In the “circular” differentiation model (Figure 1A), antigen-activated CD8^+^ T cells first acquire effector properties. A portion of these cells then dedifferentiates into Tscm or other memory subsets, while the most differentiated cells progress toward terminal effector fates and undergo apoptosis during the contraction phase.^6^^,^^27^

In the developmental/linear differentiation model (Figure 1B), activated naive T cells typically follow a unidirectional trajectory. They gradually lose their stem-like and memory characteristics while acquiring effector functions, moving through stages of Tscm → Tcm → Tem → Teff cells, with the depth of differentiation influenced by the magnitude and duration of T cell receptor (TCR) and co-stimulatory signals.^4^

Finally, the asymmetric division-driven model (Figure 1C) posits that the first division of an activated naive T cell creates daughter cells with different fates. The proximal daughter cell, which receives stronger signals, is more likely to adopt an effector-biased fate. In contrast, the distal daughter cell is biased toward a memory-like fate, from which Tscm and other long-lived memory subsets can be derived.^28^ These simplified models provide a framework for interpreting experimental data on Tscm cell ontogeny, while underscoring that multiple routes may coexist depending on antigen context and inflammatory cues.

Elegant fate-mapping studies using bromodeoxyuridine (BrdU) incorporation in acute lymphocytic choriomeningitis virus (LCMV) infection showed that long-lived memory CD8^+^ T cells arise very early after priming, from cells that divide only a limited number of times and retain a less-differentiated, stem-like profile, whereas late-dividing cells undergoing extensive proliferation are more likely to acquire terminal effector phenotypes and to be lost during the contraction phase.^29^ Recent work has identified comparable CD62L^hi^ TCF1^+^ stem-like CD8^+^ T cell populations arising during the effector phase of acute infection, which depend on inhibitory receptor signaling and preferentially seed long-term memory, further supporting an early specification of the stem-like branch.^11^^,^^12^ Longitudinal BrdU labeling after YF-17D vaccination in humans likewise demonstrated that CD8^+^ T cells incorporating the label during the early expansion phase give rise to a stable pool of long-lived, stem cell-like memory cells.^6^ Together, these observations indicate that stem-like memory characteristics are established while antigen is still present but under conditions of limited stimulation and align with data from Gattinoni and colleagues showing that Tscm cells can develop directly from naive T cells experiencing relatively weak or brief stimulation before acquiring clear effector features, positioning Tscm cells at the initial stage of a hierarchy progressing from naive T cells to Tscm, then to Tcm, Tem, and finally Teff cells.^4^^,^^30^

### Anatomical niche and environmental cues for Tscm cell stemness

The superior persistence and stemness of CD8^+^ Tscm cells are critically dependent on their anatomical localization within the secondary lymphoid organs.^9^^,^^19^ Unlike terminally differentiated effectors, Tscm cells are retained in the blood (2%–3% of total circulating T cells) and secondary lymphoid organs (2%–5% of lymph node-resident T cells), maintaining their location via homing receptors like CCR7 and CD62L.^19^ They preferentially localize within the T cell zones (paracortex) of lymph nodes, a specialized niche that actively supports their stemness properties.^19^ Within the paracortex, fibroblastic reticular cells produce interleukin-7 (IL-7) and other homeostatic factors crucial for their long-term survival, TCF1 expression, and anti-apoptotic signaling.^31^ At the same time, the chemokine environment dictates T cell fate; CD8^+^ Tscm cell differentiation is favored by retention in the CXCL9-rich paracortical regions (associated with cDC1), whereas migration to the CXCL10-rich interfollicular areas promotes effector differentiation.^19^ Furthermore, the lymphoid environment shapes the timing of innate inflammation, as transient IFN-I signaling early after priming influences the balance between effector and stem-like fates. In mouse models, short-term IFNAR blockade (within the first 24–48 h) increases the frequency of TCF1^+^ stem-like CD8^+^ T cells and improves memory formation during specific infections and mRNA-lipid nanoparticle (LNP) vaccination. However, because these interventions also prolong antigen persistence and reshape the inflammatory milieu, they do not allow a clear conclusion about a direct, cell-intrinsic effect of type I IFN on Tscm cell differentiation.^32^ Live-attenuated YF vaccination provides a well-defined *in vivo* context in which long-lived, stem-like CD8^+^ memory arises despite induction of type I IFNs, but with a delayed and tightly regulated systemic IFN-I response.^33^

Together, these findings support that the specialized anatomical niche provides homeostatic and differentiation-restraining cues. It also shapes the timing and level of inflammation, creating a favorable window for Tscm cell imprinting and long-term immune surveillance.

### Metabolic programming: The engine of Tscm cell stemness and longevity

**Figure 2:**
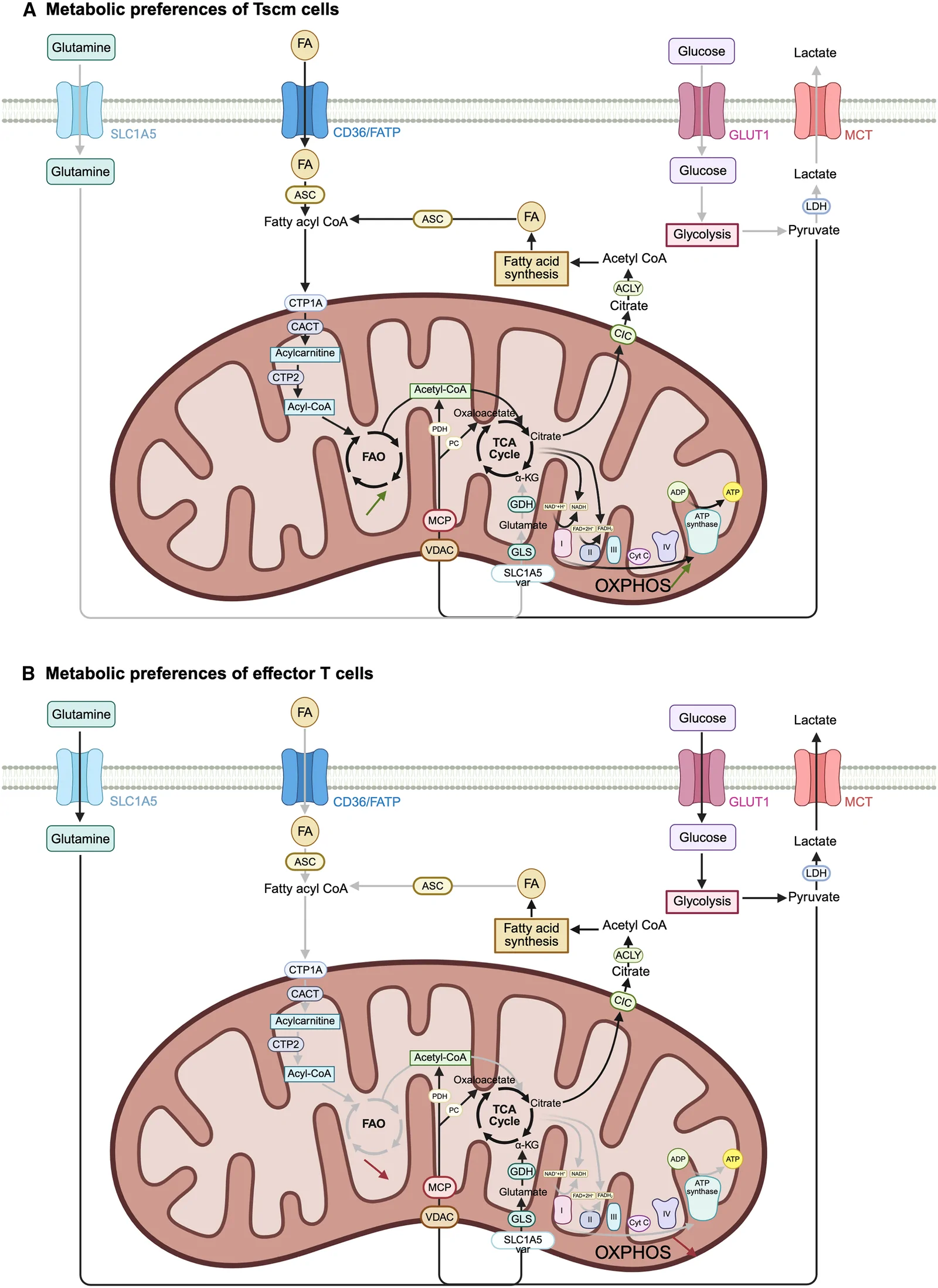
Metabolic pathways in CD8^+^ Tscm cells Comprehensive diagram showing the metabolic preferences of Tscm cells (A), including enhanced FAO, OXPHOS, and mitochondrial biogenesis, in contrast with the glycolytic preferences of effector cells (B). Key enzymes and regulatory pathways are highlighted.

The specific localization of Tscm cells is coupled with a metabolic program that differs from that of effector T cells but shares key features with other memory T cell subsets. Unlike highly glycolytic effector T cells that rely on rapid energy production, Tscm cells preferentially utilize oxidative phosphorylation (OXPHOS) and fatty acid oxidation (FAO), metabolic programs associated with longevity and stemness (Figure 2).^34^^,^^35^^,^^36^ This reliance on mitochondrial respiration is not merely a consequence of their quiescent state but actively maintains their stemness properties. Key to this is the high expression of the transcriptional coactivator PGC-1α, a master regulator of mitochondrial biogenesis that promotes mitochondrial function and stemness-associated gene expression.^34^^,^^37^ This metabolic profile grants Tscm cells an enhanced spare respiratory capacity, improved stress resistance, and reduced production of reactive oxygen species (ROS), which is crucial for long-term survival.^22^^,^^34^^,^^35^ This metabolic preference establishes the mTOR pathway as a central checkpoint for T cell fate, integrating signals from nutrients, growth factors, and energy status. The two mTOR complexes exert opposing effects on Tscm cell generation: mTORC1 activation promotes glycolysis, driving differentiation toward effector fates, while mTORC2 supports oxidative metabolism and stem cell maintenance.^38^ Consequently, pharmacological manipulation of mTOR signaling is highly effective in enhancing Tscm cell generation, as demonstrated by the transient inhibition of mTORC1 by rapamycin, which promotes Tscm cell formation *in vitro* and *in vivo*.^39^^,^^40^

Having defined the anatomical niche, metabolic profile, and required homeostatic cytokine environment of the CD8^+^ Tscm cell subset, the final key determinant of their fate is the nature of the activating signal itself. The outcome of T cell differentiation is essentially governed by the strength and duration of the initial TCR engagement, which acts as the critical switch controlling the subsequent molecular and metabolic programming.

### TCR signal strength governing Tscm cell induction

**Figure 3:**
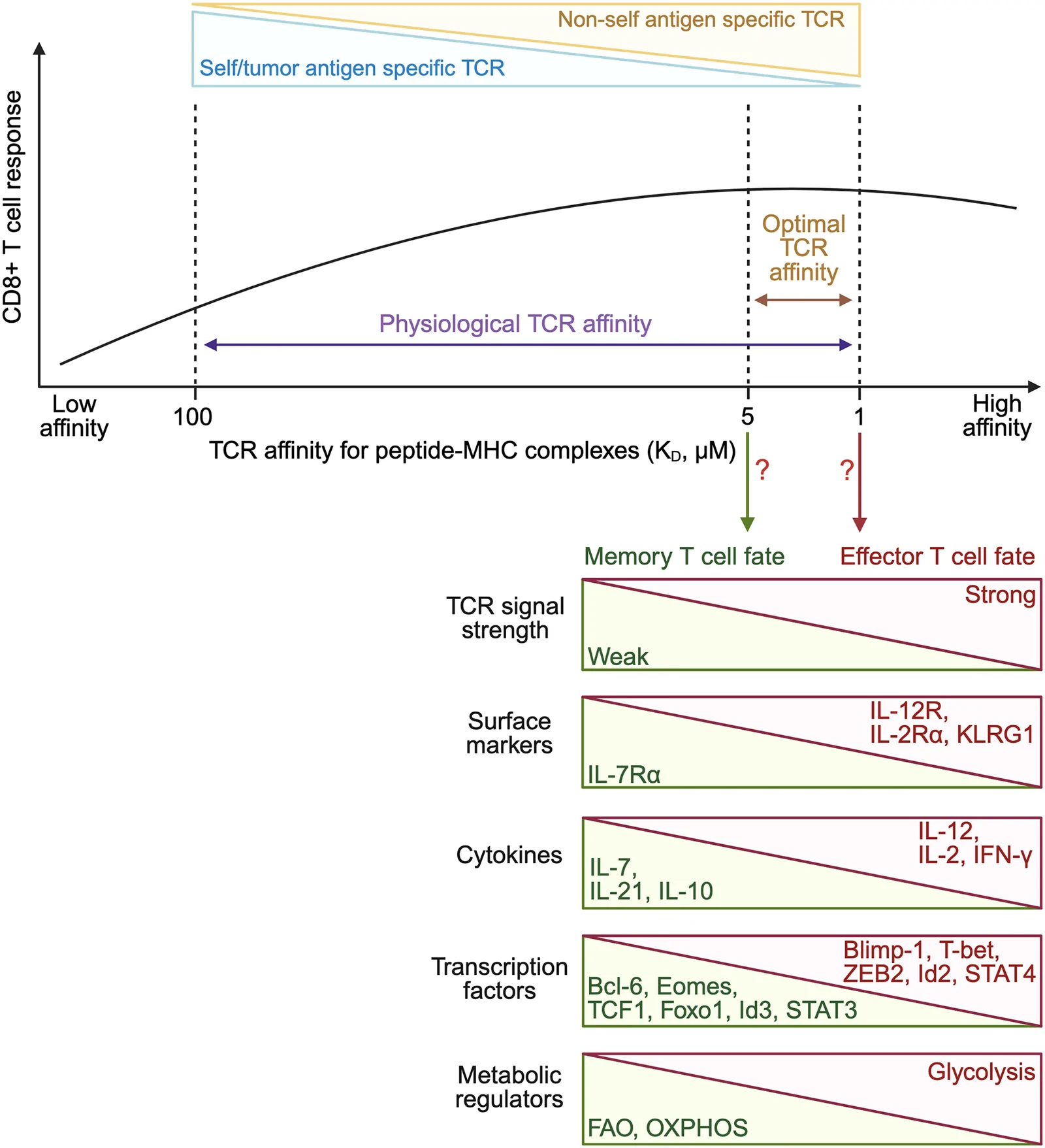
TCR affinity, antigen expression dynamics, and key factors for effector and memory CD8^+^ T cell fate CD8^+^ T cells express TCRs specific for self, tumor, or non-self-antigens, with physiological affinities for peptide-MHC ligands typically in the micromolar range (roughly 1–100 μM). Within this spectrum, affinities in the low-micromolar range are often associated with the most efficient CD8^+^ T cell responses to non-self-antigens, and further affinity increases above a threshold of about 5–10 μM generally do not enhance T cell function. Effector and memory CD8^+^ T cell fates are shaped by the strength and duration of TCR signaling together with co-stimulation, inflammatory cytokines, and the coordinated regulation of transcription factors and metabolic pathways.

**Figure 4:**
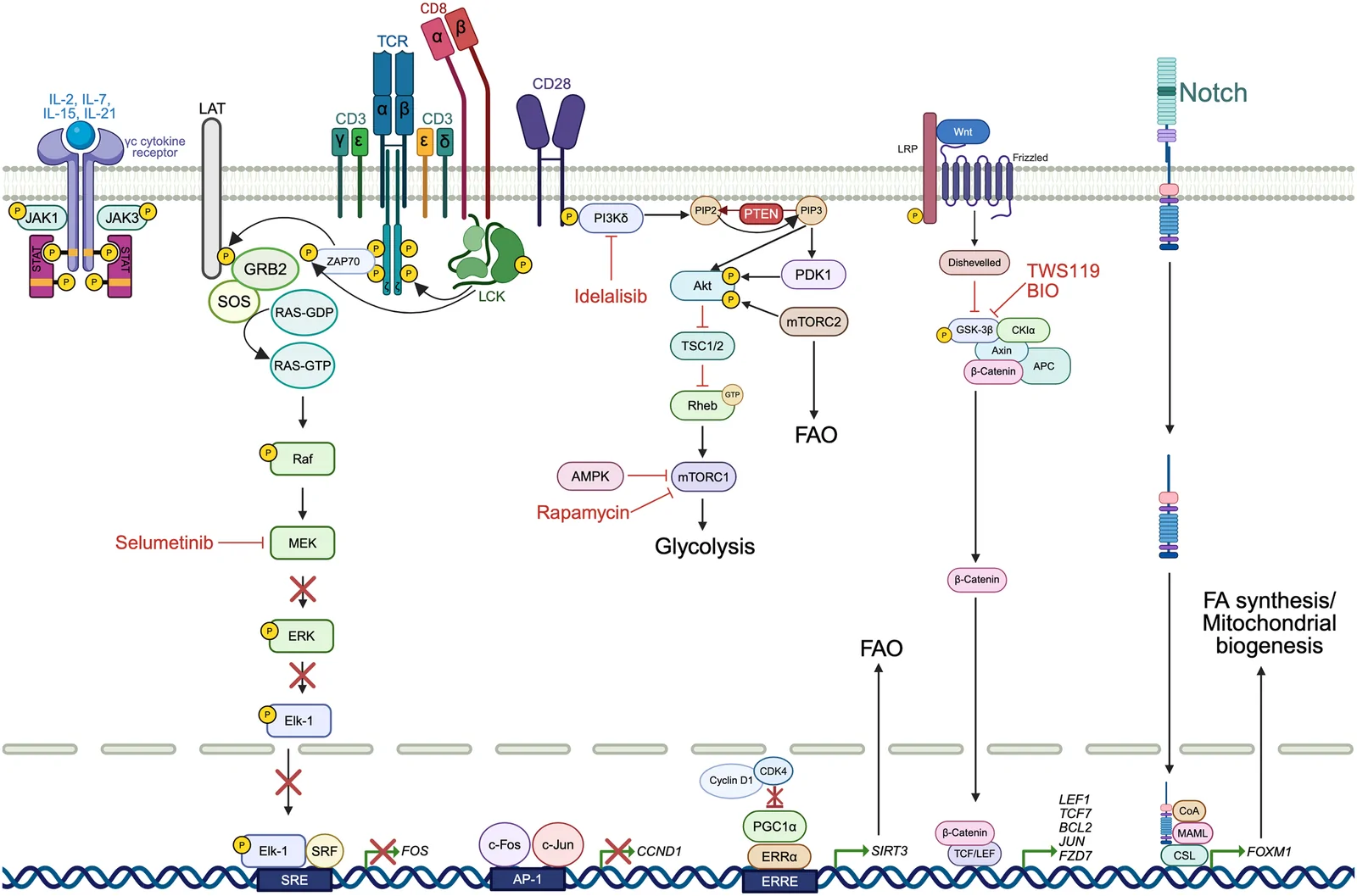
Comprehensive signaling network map for CD8^+^ Tscm cell induction Central network diagram showing the integration of TCR signaling, costimulatory pathways, cytokine signals, and metabolic cues. Key nodes include Wnt/β-catenin, mTOR, MAPK, and Notch pathways, with therapeutic targets highlighted.

The strength of TCR signaling is a key factor in determining the fate of CD8^+^ T cells. Tscm and other stem-like memory cells are preferentially generated at intermediate stimulation levels.^41^ This relationship endorses a kind of “Goldilocks principle”: signals that are too weak do not sustain activation, while excessively strong or prolonged TCR engagement can lead to terminal effector differentiation, exhaustion, or a progenitor-exhausted state, which is not conducive to forming durable stem-like memory (Figure 3).^12^^,^^34^ At the signaling level, low-to-intermediate TCR signaling maintains high levels of the phosphatase PTEN, which inhibits full PI3K/AKT activation by reducing PIP3 levels (Figure 4).^42^ This results in limited AKT activation, with phosphorylation occurring primarily at the Thr-308 site by PDK1 rather than full activation via mTORC2-mediated Ser-473 phosphorylation (Figure 4).^42^ The resulting moderate AKT activity is sufficient to promote survival and memory formation without triggering the excessive metabolic activation that drives differentiation toward effector fates,^21^ directly linking TCR signal strength to the metabolic profile described above (Figure 3). In addition to PI3K-AKT-mTOR, the classical RAS-RAF-MEK-ERK pathway downstream of the TCR also contributes to this balance. Verma et al. showed that pharmacological MEK1/2 inhibition can reprogram human and murine CD8^+^ T cells into regenerative Tscm cells with a naive-like phenotype, improved self-renewal, broader differentiation potential, and better antitumor activity.^34^ In that setting, MEK inhibition slows cell-cycle progression and dampens ERK-driven effector transcriptional program, while at the same time promoting mitochondrial biogenesis and FAO, resulting in a metabolic and epigenetic state close to Tscm cells.^34^ Importantly, these effects are achieved without shutting down TCR-mediated activation, aligning with the notion that “toning down” MAPK signaling, rather than completely abolishing it, can redirect strongly stimulated CD8^+^ T cells back toward a stem-like memory trajectory.

*In vivo*, Johnnidis et al. showed that stimulation with subdominant LCMV peptides, which deliver weaker TCR signals, preferentially generated CD62L^+^ TCF1^+^ early CD8^+^ T cell memory precursors with stem-like properties and superior long-term recall capacity compared with cells primed by dominant epitopes.^12^ In the same study, increasing the number of transferred P14 cells, which enhances intra-clonal competition and limits access to antigen, also enriched the pool of CD62L^hi^ TCF1^+^ early memory subset.^12^ This supports the notion that a decrease in effective TCR signal strength favors the formation of stem-like memory cells. These data directly link peptide affinity/avidity and antigen availability to the emergence of Tscm-like and related stem-like CD8^+^ memory cells.

Preclinical evidence, particularly in cancer immunotherapy, supports that T cells with weaker TCR signaling exhibit superior anti-tumor activity and persistence.^41^

Beyond signal intensity, the duration of TCR engagement and the dynamics of antigen presentation by dendritic cells (DCs) also play a role in determining whether T cells develop into memory cells or effector cells.^43^ Brief interactions with antigen-bearing DCs tend to promote the generation of memory precursors, while prolonged T cell-DC interactions promote continued proliferation, terminal effector differentiation, and loss of memory potential.^44^ In chronic infection settings, persistent high-intensity TCR signaling, together with high antigen load, leads to a significant upregulation of inhibitory receptors such as PD-1 and LAG-3. This environment favors the accumulation of terminally exhausted cells.^45^ In contrast, lower or more transient signals are generally associated with the maintenance of TCF1^+^ progenitor-exhausted (Tpex) cells, which retain stem-like properties and the ability to respond to future antigen encounters.^44^

Overall, these observations indicate that several factors including peptide-MHC affinity and avidity, the density and number of antigen-bearing DCs, and the persistence of antigen load collectively shape the quantity and timing of TCR signaling. This signaling further governs whether CD8^+^ T cells adopt Tscm/stem-like memory fates or progress toward terminal effector or exhausted states. These findings are of key importance for therapeutic design, indicating that optimizing the antigen dose, presentation context, and adjuvant selection is essential to achieve the precise TCR signal strength required to induce durable Tscm cells.

### Context-dependent roles of Wnt/β-catenin in CD8^+^ T cell memory and Tscm cells

The Wnt/β-catenin signaling pathway has emerged as a regulator of stem-like properties in mature CD8^+^ T cells, and its activation can strongly bias activated T cells toward a Tscm-like fate (Figure 4).^4^^,^^10^ However, *in vivo* loss-of-function mouse studies have shown that β-catenin is dispensable for CD8^+^ T cell effector differentiation as well as bulk memory formation and recall responses under physiological conditions, arguing against Wnt/β-catenin as an obligatory, stand-alone “master” pathway for CD8^+^ T cell memory.^46^^,^^47^ These loss-of-function studies did not specifically resolve Tscm cells as a distinct subset and, therefore, do not exclude context-dependent mechanisms whereby Wnt/β-catenin contributes to stem-like memory features. Indeed, in settings where Wnt/β-catenin is engaged, the weak TCR signaling discussed in the previous section facilitates this process: the resulting low-level AKT activity prevents the complete disassembly and activation of the β-catenin destruction complex, thereby promoting the necessary stabilization of β-catenin (Figure 4).^48^^,^^49^ This link ensures that only cells receiving sub-maximal activation translate that signal into a durable memory phenotype. In this stabilized state, β-catenin enables its nuclear translocation and interaction with TCF1/LEF1 transcription factors. The resulting β-catenin/TCF1 complex orchestrates a comprehensive stemness program including genes for self-renewal (*Tcf7* and *Lef1*), survival (*Bcl2*), and metabolic reprogramming (*Jun* and *Fzd7*),^49^ while actively suppressing terminal effector differentiation programs (Figure 4). Pharmacological activation of Wnt signaling, through GSK3β inhibition (e.g., TWS119 or 6-bromoindirubin-3′-oxime), has proven highly effective in generating CD8^+^ Tscm cells both *in vitro* and *in vivo*,^10^^,^^20^ positioning GSK3β inhibitors as potential vaccine adjuvants or components of adoptive cell therapy protocols (Figure 4).

### Co-stimulatory networks and receptor signaling: Refining the differentiation fate

While TCR affinity and antigen load greatly impact the fate of CD8^+^ T cells, this signaling is finely tuned by external co-stimulatory and co-inhibitory networks (Figures 3 and 4). These receptors act as crucial rheostats, modifying the amplitude and quality of the initial TCR signal to ensure the correct differentiation trajectory.

Canonical co-stimulatory signals delivered through CD28 can be either required or largely dispensable for memory CD8^+^ T cell induction, depending on the type of infection.^50^ In preclinical LCMV and vaccinia virus infection models, the CD28-CD80 pathway is not strictly required for the generation and maintenance of short- or long-term CD8^+^ memory T cells, although the magnitude of the response is reduced in its absence.^50^ In contrast, CD28-CD80 signaling is required to generate optimal CD8^+^ T cell responses to influenza and vesicular stomatitis virus infection, highlighting that co-stimulatory requirements for immune memory vary with the pathogen context, partly through the prevention of T cell anergy. Additionally, signals delivered by TNF receptor superfamily members, including 4-1BB (CD137) and OX40 (CD134), can enhance the survival and metabolic fitness of activated CD8^+^ T cells, thereby supporting the formation and maintenance of long-lived memory populations. These co-stimulatory pathways primarily promote effector and effector-memory differentiation, but by sustaining metabolically fit, they can, in a context-dependent manner, contribute to the maintenance of stem-like subsets such as Tscm cells rather than directly instructing their differentiation.^51^^,^^52^ Conversely, co-inhibitory receptors like PD-1 and CTLA-4 act as tunable brakes on TCR and costimulatory signaling rather than simply inhibiting the formation of Tscm cells.^12^ Transient expression of PD-1 in early antigen-experienced precursors can limit terminal effector differentiation and has been associated with supporting stem-like T cell progenitors that have Tscm/Tcm cell potential.^12^ In contrast, chronic PD-1 engagement leads to metabolic insufficiency, restricts central memory differentiation and promotes terminal exhaustion, thereby depleting the Tscm-like compartment.^52^^,^^53^ Another finding is that the differentiation of Tscm cells is directly influenced by two known receptor-ligand systems. First, the interaction between CD40 on antigen-presenting cells (APCs) and CD40L on T cells provides a powerful co-stimulatory signal that optimally promotes Tscm cell induction.^54^ This is a direct link, as CD40 signaling is recognized for its capacity to stabilize β-catenin and activate the Wnt pathway in responding T cells, thereby providing the necessary transcriptional signal for stemness.^54^ Second, the Notch signaling pathway is another key regulator of CD8^+^ T cell fate. While several studies indicate that Notch promotes short-lived effector differentiation and PI3K-AKT activation *in vivo*, other work has shown that, under specific experimental conditions, Notch can cooperate with Wnt/β-catenin to sustain TCF1-driven transcriptional programs and generate induced Tscm-like cells from activated T cells.^35^^,^^36^^,^^55^^,^^56^^,^^57^ Overall, these findings suggest that the impact of Notch on Tscm versus terminal effector differentiation is highly context and dose dependent, rather than reflecting a universal requirement for Notch in Tscm cell survival and self-renewal.

This signaling cascade is also influenced by the local cytokine environment. IL-7 and IL-15 are essential for Tscm cell homeostasis,^4^^,^^58^^,^^59^ while IL-21 acts as a powerful differentiation factor.^60^^,^^61^^,^^62^^,^^63^^,^^64^^,^^65^ IL-21 plays a critical role in promoting robust cytotoxic T lymphocyte responses and in sustaining the function and persistence of memory CD8^+^ T cells, particularly under conditions of chronic or repeated antigen exposure.^60^^,^^61^^,^^62^^,^^63^^,^^64^^,^^65^ Mechanistically, IL-21 signals through STAT3 to induce and maintain transcription factors such as BATF and Blimp-1, often in cooperation with IRF4, thereby driving effector differentiation and cytotoxic function, while simultaneously preserving CD8^+^ T cell persistence and preventing progression to deeply exhausted, terminally dysfunctional states (Figure 2).^63^^,^^64^^,^^65^ In this way, IL-21 associates the induction of effector differentiation programs with the preservation of durable memory potential, instead of simply acting as a pro-exhaustion cytokine. In addition, and as mentioned earlier, type I IFN signaling exerts a critical early influence on CD8^+^ T cell fate. Transient blockade of IFNAR signaling during the first days of the response has been shown to favor the emergence of stem-like CD8^+^ T cells and to limit premature exhaustion, although these effects likely reflect a combination of direct actions on T cells and indirect changes in antigen load and the inflammatory microenvironment.^32^ This inhibitory effect is reinforced by the observation that the YF vaccine, a potent CD8^+^ Tscm cell inducer, elicits a delayed systemic IFN-I production (peaking days 3–7).^33^ This timing-dependent antagonism highlights how the acute innate response must be carefully managed to prevent the premature terminal differentiation of T cell precursors.

### Transcriptional integration of Tscm cell fate: TCF1, FOXO1, and T-bet in infection versus vaccination

At the transcriptional level, TCF1 (*Tcf7*) serves as a central hub for Tscm and other stem-like CD8^+^ T cell subsets. However, its activity is integrated with that of additional transcription factors, whose contributions depend on the inflammatory and antigenic context. In infection models, FOXO1 is primarily associated with the maintenance of stem-like and central memory fates.^66^ In contrast, T-bet is classically linked to short-lived effector differentiation (Figure 3). The loss of FOXO1 selectively impairs memory formation, while T-bet deficiency mainly affects terminal effector cells.^66^ Recent studies using adjuvanted subunit vaccination show that effective CD8^+^ T cell responses to vaccines rely on both FOXO1 and T-bet.^66^ A deficiency in either factor compromises not only effector responses but also the generation of memory cells.^66^ Single-cell RNA sequencing in this context reveals simultaneous transcriptional activity of T-bet, FOXO1, and TCF1 in vaccine-elicited CD8^+^ T cells undergoing clonal expansion.^66^ This combination is not observed to the same extent during *Listeria* infection, indicating that a less inflammatory, tightly controlled vaccine environment allows the cooperative engagement of effector- and memory-associated transcriptional programs.^66^ These findings support the view that Tscm and related stem-like CD8^+^ memory cells induced by vaccination are not identical to those generated by natural infection. Instead, they represent context-dependent expressions of a common stem-like program, in which TCF1, FOXO1, and T-bet are differentially balanced based on factors such as antigen load, adjuvant type, and the inflammatory environment. In other words, Tscm-like cells elicited by adjuvanted vaccination appear to rely on a coordinated FOXO1-TCF1-T-bet module, whereas infection-elicited stem-like cells emerge under conditions where FOXO1-TCF1 activity is more clearly opposed by high-level T-bet expression.

In rational therapy, achieving the right balance and timing of signals from various networks is essential. An optimal environment with co-stimulatory signals and cytokines maintains the appropriate strength of TCR signaling and supports stem-like memory programming. This balance prevents T cells from becoming overly activated, which can lead to short-lived effector fates. Strategically manipulating co-stimulatory pathways, receptor-ligand interactions, and cytokine signals, while adjusting inflammatory conditions, is key to designing effective vaccines that induce Tscm cells. Additionally, optimizing CAR T cell products to promote long-lasting memory instead of terminal differentiation is also a key objective.

## CD8^+^ Tscm cells in human diseases

The study of CD8^+^ Tscm cells is important in viral infections and cancers because these cells are essential for long-term immunity against persistent threats.

### Viral infections: Lessons from acute and persistent pathogens

In the context of viral pathogens, CD8^+^ Tscm cells occupy the apex of the memory T cell hierarchy, serving as a key long-term reservoir whose proliferative and multi-differentiation potential is central to both the rapid clearance during acute infection and the sustained control often compromised during viral chronicity.

### Acute infection: COVID-19

The recent COVID-19 pandemic, caused by SARS-CoV-2, has provided a critical and timely model to investigate the role of CD8^+^ Tscm cells in the formation of durable protective immunity following an acute viral infection. Indeed, convalescent patients demonstrate robust generation of SARS-CoV-2-specific CD8^+^ T cells with stem cell-like memory features, with frequencies correlating positively with symptom-free days and negatively with disease severity.^67^ Longitudinal studies reveal that SARS-CoV-2-specific CD8^+^ Tscm cells persist for at least 10–12 months after infection, maintaining their phenotypic and functional characteristics.^68^^,^^69^

Interestingly, studies on SARS-CoV-2 have revealed that patient age critically influences the generation and maintenance of CD8^+^ Tscm cells, suggesting that immunosenescence significantly affects the long-term quality of antiviral memory. Indeed, following SARS-CoV-2 infection, older individuals show reduced frequencies of Tscm cells despite comparable overall T cell responses.^70^

### Chronic infections: LCMV, HIV, CMV, HCV, and EBV

#### The LCMV model of infection

In addition to their immediate responses, CD8^+^ T stem cell-like cells play a crucial role in chronic viral infections. The conceptual framework for stem-like CD8^+^ T cells in chronic infection was initially established in the murine LCMV model. In this context, several research groups identified a population of PD-1^+^ TCF1^+^ CXCR5^+^ “stem-like” virus-specific CD8^+^ T cells that reside in lymphoid tissues, undergo slow self-renewal, and continuously produce more differentiated, terminally exhausted progeny.^24^^,^^71^^,^^72^ These stem-like cells emerge early during chronic infections and are essential for sustaining the antiviral CD8^+^ T cell response over time. They serve as the primary proliferative reservoir upon PD-1/PD-L1 blockade, facilitating the functional rescue of exhausted T cells and enhancing viral control.^71^^,^^72^

Clonal tracking and TCR repertoire analyses have further demonstrated that the exhausted T cell compartment is almost entirely derived from this stem-like subset, highlighting its role as the upstream progenitor population during chronic LCMV infection.^24^^,^^25^

These pioneering studies by Ahmed and colleagues identified a pool of stem-like, progenitor-exhausted CD8^+^ T cells that maintain long-term immunity against persistent antigens. Their work provided the foundation for subsequent research on similar cell populations in chronic human infections, including human immunodeficiency virus (HIV), cytomegalovirus (CMV), hepatitis C virus (HCV), and Epstein-Barr virus (EBV).

#### HIV-1 infection

To analyze and understand the role of Tscm cells in the context of HIV chronic infection, it is crucial to differentiate between CD4^+^ and CD8^+^ Tscm cells. CD4^+^ Tscm cells express CCR5 and have low levels of HIV restriction factors, making them permissive to HIV with R5 tropism, which in turn enables them to function as viral reservoirs.^73^

Evidence related to CD8^+^ Tscm cells is inconclusive regarding their role in controlling HIV-1 infection, with conflicting results reported in the literature. The frequencies and absolute numbers of total or specific CD8^+^ Tscm cells, as indicated by Ribeiro S. P. et al., do not differ among elite controllers and progressors, and both groups exhibit lower frequencies of total CD8^+^ Tscm cells than uninfected individuals.^74^ A recent study by Rachel L. Rutishauser et al. reported that elite controllers have a higher proportion of HIV-specific CD8^+^ T cells expressing TCF1 than non-controllers, regardless of the antiretroviral therapy (ART) status.^75^ Studies of untreated patients found no significant correlation between the quantities of circulating CD8^+^ Tscm cells and the control of viral load.^74^^,^^76^

Apart from that, the frequencies of specific CD8^+^ T cells with stem cell-like properties and CXCR5 homing characteristics are significantly higher in seronegative women exposed to HIV than in unexposed seronegative women, progressors, or HIV-infected women undergoing ART.^77^^,^^78^

In non-controller-infected patients, the recovery of CD8^+^ Tscm cell levels after the initiation of ART shows inconsistencies across studies. For instance, Vigano et al. found that patients undergoing ART had significantly more total or Th1-cytokine-secreting specific CD8^+^ Tscm cells than elite controllers or progressors.^76^ Conversely, Utay et al. reported no differences in the levels of circulating CD8^+^ Tscm cells between progressors and patients treated with ART, whether in the acute or chronic phase of infection.^79^

In HIV-infected patients who successfully restored their CD8^+^ Tscm cells while on ART, the duration of therapy is associated with a gradual restoration of functional HIV-specific Tscm cells.^76^ Furthermore, starting ART earlier contributes to more effective restoration. A longitudinal study of HIV-infected patients, conducted before and 1 month or 1 year after ART, showed a significant decrease in exhausted CD8^+^ Tscm cells at the later time point.^80^

Patients who interrupted ART exhibited significantly higher frequencies of exhausted CD8^+^ Tscm cells compared with those who maintained consistent treatment.^81^

Recent breakthrough studies have demonstrated that HIV-specific CD8^+^ T cells can be reprogrammed toward Tscm phenotypes through GSK3β inhibition, resulting in enhanced antiviral activity and improved persistence.^20^ This reprogramming involves signaling, metabolic, and gene expression changes characteristic of stemness, including reduced mTORC1 signaling and quiescent metabolism, providing proof of concept for therapeutic Tscm cell enhancement in humans.

#### CMV infection

CMV infection is a benign, persistent infection except in immunocompromised individuals and infants infected before birth.^82^ In industrialized countries, 60%–70% of adults have been infected with CMV.^83^ After a typically self-limiting, subclinical primary infection, CMV persists in leukocytes in a latent form for life, thanks to the host immune response that stops CMV replication and dissemination. CMV can reactivate when the immune system is weakened.^84^^,^^85^ A limited number of studies have investigated CD8^+^ Tscm cells in CMV infection, but none have focused on the acute phase of the infection. Svetlana Di Benedetto et al. did not observe an increase in total CD8^+^ Tscm cells among CMV-seropositive young women or men when compared with their respective seronegative control groups. The frequencies of total CD8^+^ Tscm cells were not altered in old CMV-seropositive individuals of both sexes, in contrast to other long-lived memory or terminally differentiated Temra CD8^+^ T cell populations.^86^ Central memory CD8^+^ T cells decrease with age in seropositive men.^86^ Terminally differentiated Temra CD8^+^ T cells were found to be significantly increased in older seropositive men and women compared with their younger seropositive counterparts and older seronegative individuals.^86^ Additionally, higher expression of the CD57 senescent marker on CD8^+^ T cells is characteristic of older CMV-seropositive men and women.^86^

In their study, Schmueck-Henneresse et al. demonstrated that CMV-specific CD8^+^ Tscm cells retain their ability to proliferate, produce cytokines, and exhibit cytotoxic functions.^87^ Within the pool of CMV-specific memory CD8^+^ T cells, both Tscm and non-Tscm cells share a similar TCR repertoire.^86^

#### HCV infection

HCV can cause both acute and chronic infection of the liver.^88^ Patients infected with HCV who have not received treatment and have a median duration of infection of 3 years show an increase in CD8^+^ Tscm cells compared with seronegative individuals.^89^ The proportion of CD8^+^ Tscm cells correlates positively with the CD8^+^ Tcm lymphocytes and negatively with effector CD8^+^ T cells. In patients infected with HCV, a higher frequency of CD8^+^ Tscm cells was associated with lower viral loads and reduced expression of chronic activation markers on lymphocytes. Thus, CD8^+^ Tscm cells might be linked to the control of HCV infection.

#### EBV infection

The EBV is one of the most common human viruses, and most individuals are unaware of their infection.^90^ The role of CD8^+^ Tscm cells in controlling EBV has mainly been investigated in the context of post-transplant lymphoproliferative disorder (EBV-PTLD). This life-threatening condition can arise after hematopoietic stem cell transplantation or solid organ transplantation.^91^ EBV-PTLD typically occurs as a result of immunosuppressive drugs that are used to prevent transplant rejection and allow the reactivated EBV to cause uncontrolled proliferation of B cells.^91^ The treatment approaches are multifaceted and focus on restoring the host’s immune responses, particularly allowing the patient’s own T cells to regain function and control the EBV-infected cells. One therapeutic option is the adoptive cellular therapy where patients’ EBV-specific T cells are collected, specifically trained *ex vivo* to recognize and eliminate EBV-infected B cells, and reinfused into the original donor patient. In the context of this *ex vivo* training of CD8^+^ T cells, EBV-specific CD8^+^ Tscm cells have been reported to provide more durable control of EBV-transformed B cells than conventional memory T cell subsets, owing to their superior persistence, self-renewal capacity, and sustained ability to generate cytotoxic progeny, rather than to higher immediate cytotoxic activity per cell. Additionally, these Tscm cells exhibit enhanced proliferative responses to IL-7 stimulation.^55^ Furthermore, in humanized mouse models, the adoptive transfer of EBV-specific Tscm cells generated *in vitro* from human peripheral lymphocytes provides superior long-term control of EBV-driven lymphoproliferative disease compared with the transfer of other memory T cell subsets.^92^

In the end, the study of CD8^+^ Tscm cells across various viral infections, from acute to chronic, provides compelling evidence of their critical role in long-term immunity, highlighting their potential as targets for therapeutic interventions and personalized medicine.

### Cancer: The double-edged sword

Given their unparalleled capacity for long-term persistence and self-renewal, the focus on CD8^+^ Tscm cells naturally extends from controlling chronic infections to optimizing anti-tumor immunity, where harnessing their characteristics is central to advancing adoptive cell therapies, particularly chimeric antigen receptor (CAR) T cell treatments.

The role of CD8^+^ Tscm cells in cancer immunity is complex, as these cells can have both protective and potentially detrimental effects depending on the context. Multiple cancer types demonstrate positive correlations between tumor-infiltrating CD8^+^ Tscm cell frequencies and patient prognosis. In HPV-infected individuals, HPV-specific CD8^+^ Tscm cells are more prevalent in patients without HPV-related oropharyngeal tumors compared with those with established malignancies, suggesting a protective role in preventing viral oncogenesis.^54^ These cells maintain proliferative capacity and can differentiate into functional effector cells upon antigen encounter. Colorectal cancer studies have revealed that TCF1^+^ PD-1^+^ Tpex CD8^+^ T cells correlate with improved responses to checkpoint inhibitor therapy.^93^ Mechanistic and translational studies indicate that these TCF1^+^ PD-1^+^ stem-like cells act as the main source of renewed effector CD8^+^ T cells following PD-1/PD-L1 blockade, underscoring the central role of the Tpex compartment, rather than classical Tscm, in mediating anti-tumor reinvigoration in this setting. More broadly, across multiple solid tumors, intratumoral and lymph-node-resident TCF1^+^ PD-1^+^ stem-like CD8^+^ T cells have been identified as the key population expanded by immune checkpoint blockade. Their abundance before therapy predicts clinical benefit, further establishing Tpex rather than terminally exhausted cells as the principal targets and effectors of PD-1-based immunotherapies.^94^^,^^95^^,^^96^^,^^97^^,^^98^

The identification of Tscm cells as the most durable and multipotent memory T cell subset provides a clear mechanistic rationale for their therapeutic exploitation in oncology. Therefore, considerable research efforts are now directed at enriching or engineering the Tscm phenotype within adoptive cell products, forming the basis for improving the efficacy and persistence of CAR T cell therapies. Indeed, the therapeutic efficacy of anti-tumoral CAR-T cells relies on their ability to proliferate and survive after their re-infusion into patients.^99^ Research has shown that both CD8^+^ Tscm and Tcm cells persist longer and exhibit superior anti-tumor activities compared with CD8^+^ Tem cells.^100^ A transcriptomic analysis of patients with chronic lymphocytic leukemia who were treated with CAR-T cells found that the proportions of Tscm and Tcm cells were higher in patients who achieved a complete or partial response compared with those who did not respond.^101^ Additionally, complete responders showed persistence of CAR-T cells for over 5 years.^101^

As a result, manufacturing protocols have been developed to enhance the induction of Tscm-based CAR-T cells. These protocols typically include *ex vivo* expansion using Wnt pathway activators, along with IL-7, IL-15, and occasionally IL-21, or refining cell metabolism from glycolysis toward OXPHOS.^102^^,^^103^ A study found that a protocol designed to enrich Tscm cells significantly increased the number of CAR-Tscm cells in patients with B-cell acute lymphoblastic leukemia. This enrichment led to prolonged anti-tumor responses and improved long-term remissions.^104^

CD8^+^ Tscm cells are emerging as a central target in cancer immunology, protecting against viral oncogenesis and sustaining responses to checkpoint inhibitors and CAR T cell therapy. Researchers are focusing on enhancing the stem-like properties of stem cells to develop more effective and lasting cancer treatments.

## Therapeutic interventions for inducing Tscm cells

Given the central role of CD8^+^ Tscm cells in both viral control and tumor surveillance, we need to explore methods for actively inducing or expanding this population. Current therapeutic interventions focus on two main axes: the administration of specific pharmacological agents and the design of optimized vaccination strategies. The latter includes developing novel vaccine platforms or integrating known platforms with metabolic adjuvants, all aimed at skewing T cell differentiation toward the wanted Tscm cell phenotype.

### Pharmacological interventions

The molecular understanding of Tscm cell induction has revealed several druggable targets for therapeutic intervention (Table 2). These targets can be broadly categorized into pathway activators (promoting stemness) and pathway inhibitors (preventing terminal differentiation).

**Table 2:** Druggable targets for CD8^+^ Tscm cell enhancement

Drug	Effect	Development status	Clinical application
GSK3 inhibitor (TWS119 or BIO)	Wnt/β-catenin activation	phase 2 (various indications)	vaccine adjuvant
Rapamycin	mTORC1 downregulation	FDA approved (organ transplantation)	immunosuppressive drug
NAC	reduction of ROS production	FDA approved (chronic lung disease)	antioxidant
PI3Kδ inhibitor (idelalisib)	PI3Kδ-AKT-mTOR downregulation	FDA approved (cancer)	anti-neoplastic agent
Altered peptide ligand + IFNgR knockout or α-fetoprotein peptide	TCR weak stimulation	preclinical	T cell activation
Notch ligand	stemness signaling maintenance	preclinical	CAR-T enhancement
Anti-CD40 partial agonist Ab fused to antigens	APC targeting	phase 1/2 (vaccines)	vaccination platform
MEK1/2 inhibitor (selumetinib)	OXPHOS and FAO upregulation	FDA approved (cancer)	T cell manufacturing
	MAPK pathway downregulation		
Urolithin A	Pink1/Parkin-mediated mitophagy	phase 2–3 (cancer)	anti-cancer drug
Anti-PD-1 Ab	immune checkpoint inhibition	FDA approved (cancer)	anti-cancer drug
LDH inhibitor (NCI-737)	glycolysis downregulation	preclinical	combination therapy
IL-21	cytokine enhancement	phase 2 (immunotherapy)	vaccine adjuvant
Anti-IFNAR Ab	IFN-I pathway downregulation	preclinical	anti-inflammation

Combination approaches targeting multiple pathways simultaneously have shown synergistic effects in preclinical models. For example, combining GSK3β inhibition with IL-21 and IL-7 supplementation or MEK inhibitor with IL-2 supplementation produces superior Tscm cell generation compared with either intervention alone.^100^

### Vaccine platforms: Lessons from success and failure

The generation of robust, long-lived CD8^+^ Tscm cells through vaccination is considered the ultimate therapeutic goal for durable protection against viral infections and cancer. A comparative analysis of current vaccine platforms reveals that successful Tscm cell induction is intrinsically linked to two key parameters: the quality and persistence of the TCR signal delivered and the associated innate immune milieu within the lymphoid tissue.

#### Live-attenuated vaccines

Historically, live-attenuated vaccines (LAVs), exemplified by the YF (YF-17D) vaccine, have long served as the gold standard for inducing highly persistent CD8^+^ Tscm cells. Specific CD8^+^ Tscm cells induced by YF-17D vaccine remain detectable more than 25 years after a single dose.^5^ Longitudinal human studies show that YF-17D replication and antigenemia drive a CD8^+^ T cell response that peaks around days 10–15 and then contracts into a long-lived memory pool, providing an *in vivo* context in which durable stem-like memory is established during a prolonged, yet controlled, primary response.^105^ In this setting, YF-induced Tscm cells progressively acquire a polyfunctional profile and re-express CD45RA over time, while maintaining self-renewal and multipotent differentiation capacity upon restimulation.^5^^,^^106^

Taken together, LAVs such as YF-17D and some natural acute viral infections represent prototypical platforms capable of imprinting highly durable stem-cell-like CD8^+^ memory, but their broad use is constrained by safety and manufacturing considerations that limit how far this strategy can be generalized.

#### mRNA-based platform vaccines

From a practical standpoint, mRNA platforms offer unmatched speed and flexibility in production, but data from COVID-19 vaccination indicate that spike-specific antibody titers and T cell responses decline substantially within 6–12 months after primary vaccination, so that protection against infection progressively wanes over time and typically requires booster doses to maintain. Studies on the BNT162b2 mRNA vaccine confirm the generation of spike-specific CD8^+^ Tscm cells that persist for at least 6 months, with early Tscm cell generation correlating with overall response durability.^107^^,^^108^ Current data do not allow a definitive head-to-head comparison of absolute Tscm cell frequencies between mRNA platforms and classical LAVs in matched settings. These differences likely reflect, at least in part, fundamental disparities in antigen kinetics and innate immune activation. mRNA vaccines are characterized by a short period of antigen expression and a strong, early inflammatory response with robust IFN-I production. In contrast, LAVs such as YF-17D exhibit a more extended period of controlled viral replication and a delayed systemic IFN-I peak. As mentioned earlier, a study in mice found that temporarily blocking IFN-I signaling shortly after priming increases TCF1^+^ stem-like CD8^+^ T cells and enhances memory formation after specific infections and mRNA-LNP vaccination. These effects likely arise from both direct actions on T cells and changes in antigen persistence and the inflammatory environment.^32^ Thus, while IFN-I is essential for early antiviral defense, an excessive initial IFN-I burst may disrupt long-lasting memory formation in some vaccines, whereas a more controlled IFN-I response, as seen after YF-17D vaccination, aids in the development of durable stem-cell-like CD8^+^ memory.^32^

#### Viral vectors/DNA prime-MVA boost

Beyond live-attenuated and mRNA platforms, viral-vector and DNA prime-viral boost strategies have also been shown to induce Tscm cells. Poxvirus vectors, for instance, induce strong IFN-I and inflammasome activation,^109^^,^^110^^,^^111^ pathways that seem unfavorable for Tscm cell generation. Success in CD8^+^ Tscm cell generation appears confined to heterologous prime/boost regimens (e.g., DNA prime followed by MVA boost),^112^ suggesting that an optimized sequential, rather than a single acute, signal is key. Nevertheless, the overall magnitude of Tscm cells remains modest, and platform-specific issues such as pre-existing vector immunity and, in the case of some adenoviral vectors, rare but serious adverse events constrain dose and boosting strategies, motivating ongoing work on alternative vectors, routes of delivery, and adjuvant combinations to preferentially expand stem-like memory while maintaining acceptable safety profiles.

#### Subunit vaccines

Subunit vaccines, while generally safe, often require repeated boosting because they tend to elicit comparatively shorter-lived antibody and T cell responses than replicating platforms. In mouse models using OVA protein combined with a broad range of adjuvants (including poly(I:C), MPLA, Pam3Cys, CpG, and alum), adjuvanted subunit vaccination drives a CD8^+^ T cell response enriched in CD127^+^ TCF1^hi^ memory-phenotype cells, with higher per-cell expression of TCF1, FOXO1, and CD127 than after infection with *Listeria monocytogenes* expressing OVA.^66^ Single-cell transcriptional analyses further show that vaccine-elicited cells undergoing clonal expansion co-activate T-bet, FOXO1, and TCF1 programs, indicating that Tscm-like cells induced by adjuvanted subunit vaccines are transcriptionally distinct from infection-induced Tscm cells. In vaccination settings, the generation of long-lived memory CD8^+^ T cells is co-dependent on both effector- and memory-associated transcription factors. In this context, adjuvants that impose a strongly STAT1/STAT4-biased inflammatory environment are likely to skew this balance toward higher T-bet activity and more short-lived effectors, at the expense of optimally stem-like memory cells.^113^

The fundamental challenge across these vaccine platforms is how to sustain a sufficient antigenic signal to preserve stem-like memory potential without relying on an acute inflammatory burst that instead drives terminal effector differentiation.

#### Rational vaccine design: Optimizing CD8^+^ Tscm cell induction through signaling

This mechanistic analysis has shifted the field toward stemness-oriented vaccination and highlights the remaining scope to develop vaccine platforms that more effectively induce CD8^+^ Tscm cells. A highly compelling strategy involves the direct targeting of the CD40 receptor on APCs. Ligation of CD40 provides optimal signals for CD8^+^ Tscm cell induction,^54^ because it upregulates β-catenin, thereby directly activating the Wnt signaling pathway, which is integral to regulating the transcription factors necessary for T cell stemness.^54^ Preclinical studies using CD40 agonists demonstrated the formation of functional CD8^+^ Tscm cells with potent anti-tumor activity, confirming the Wnt/β-catenin pathway as essential for their generation in this setting.^54^ The CD40-targeting vaccine platform (e.g., CD40.CoV2) exemplifies this rational design.^114^ This approach effectively delivers the antigen directly to APCs while simultaneously providing the crucial CD40 co-stimulation. Head-to-head experiments confirmed that the CD40.CoV2 vaccine elicited a significantly higher frequency of CD8^+^ Tscm cells than the BNT162b2 mRNA vaccine, which predominantly activated effector CD8^+^ T cells.^114^ This observation strongly supports the hypothesis that optimal TCR signaling strength, sufficient for effective priming without driving terminal differentiation, is the key mechanism mediated by the activated APCs.^114^

### The future: Metabolic adjuvants and T cell fate

These adjuvants are specifically designed to tune the cellular metabolome, thereby shifting the transcriptional state to favor the metabolic and energetic profiles required for Tscm cell self-renewal and persistence over the glycolytic demand of terminal effector differentiation. Such approaches are crucial for translating the potential of Tscm cell biology into clinically relevant, long-lasting protective immunity.

The core mechanistic principle is that Tscm cells rely predominantly on mitochondrial respiration and FAO, in contrast to the glycolytic dependency of terminally differentiated effector cells. Consequently, interventions that promote FAO while limiting glycolysis effectively enhance Tscm-cell generation during priming and memory maintenance.^34^^,^^40^ Metabolic adjuvants thus represent an emerging class of vaccine components designed to tune T cell metabolism toward stemness. Compounds that either support FAO or modulate key metabolic checkpoints have shown considerable promise: the antioxidant N-acetylcysteine (NAC), which promotes FAO, enhances Tscm cell generation in preclinical models,^22^ and pharmacological activation of AMPK or inhibition of mTORC1 likewise shifts T cell metabolism toward stemness-promoting programs. Transient mTORC1 inhibition by a short course of high-dose rapamycin notably improves vaccine-induced CD8^+^ T cell memory responses in mice,^115^ and this strategy is now being evaluated clinically to boost mRNA SARS-CoV-2 vaccine efficacy in kidney transplant recipients receiving a third dose.^116^ These findings collectively emphasize that metabolic interventions are most effective when applied early, during the initial phase of T cell activation, to divert differentiation toward long-lived Tscm.

## Conclusion

The evidence accumulated over the past two decades conclusively supports CD8^+^ Tscm cells as the central mediators of durable immunity across viral infectious diseases and cancer. Their properties include self-renewal capacity, multipotency, exceptional longevity, and robust polyfunctional effector functions. These qualities make them the essential target for next-generation vaccination and adoptive immunotherapy strategies. The enduring presence of Tscm cells induced by the YF vaccine for over 25 years sets a critical gold standard that future immunological interventions must aim to replicate.

The detailed mechanistic dissection of Tscm cell induction has provided a complete roadmap for therapeutic engineering. We now understand that the generation of this subset is not just stochastic, but tightly governed by integrated checkpoints, including the low-amplitude TCR signal, the activation of stemness pathway, and an energy profile heavily reliant on FAO. Furthermore, the fate is crucially modulated by the appropriate cytokine and co-stimulatory balance (e.g., CD40/CD40L, IL-21) and the controlled, non-terminal inflammatory environment of the lymphoid niche.

To reach the goal of universal Tscm cell induction, the scientific community must transition from empirical testing to rational design. This necessitates a focus on innovative methods to deliver antigens, the development of metabolic adjuvants, or the refinement of existing adjuvants based precisely on the quality and temporal control of the inflammatory environment they create. The successful translation of this Tscm mechanistic blueprint into clinical practice represents the most profound opportunity to fundamentally enhance the efficacy and durability of protective T cell responses in the 21st century.

## Acknowledgments

This work was funded by the Vaccine Research Institute (VRI) via the grant ANR-10-LABX-77 and the IHU SEPSIS - PROMETHEUS Comprehensive Sepsis Center, Paris-Saclay University, Saclay, France, via the grant ANR ANR-23-IAHU-0004. L.N. received a PhD grant from the Graduate School “Life and Health Sciences” (ED 402) at the 10.13039/501100009411Université Paris-Est Créteil.

## Author contributions

Conceptualization and investigation, L.N., Y.L., and V.G.; writing – review, L.N., Y.L., and V.G.; editing, L.N. and V.G.; supervision, V.G.; all authors have read and agreed to the published version of the manuscript.

## Declaration of interests

The authors declare no competing interests.

## Declaration of generative AI and AI-assisted technologies in the writing process

During the preparation of this work, the authors used Grammarly’s generative AI features to enhance the English language editing of the manuscript.
